# Uncovering the mechanisms of salicylic acid-mediated abiotic stress tolerance in horticultural crops

**DOI:** 10.3389/fpls.2023.1226041

**Published:** 2023-08-28

**Authors:** Hua Yang, Rui Fang, Ling Luo, Wei Yang, Qiong Huang, Chunlin Yang, Wenkai Hui, Wei Gong, Jingyan Wang

**Affiliations:** ^1^ Provincial Key Laboratory of Forestry Ecological Engineering of Sichuan Province, College of Forestry, Sichuan Agricultural UR.A.niversity, Chengdu, China; ^2^ School of Environment, Sichuan Agricultural University, Chengdu, China

**Keywords:** poor yield, photosynthetic impairments, stomatal conductance, signaling molecules, stunted growth

## Abstract

Salicylic acid (SA) has been recognized as a promising molecule for improving abiotic stress tolerance in plants due to its ability to enhance antioxidant defense system, and promote root architecture system. Recent research has focused on uncovering the mechanisms by which SA confers abiotic stress tolerance in horticultural crops. SA has been shown to act as a signaling molecule that triggers various physiological and morphological responses in plants. SA regulates the production of reactive oxygen species (ROS). Moreover, it can also act as signaling molecule that regulate the expression of stress-responsive genes. SA can directly interact with various hormones, proteins and enzymes involved in abiotic stress tolerance. SA regulates the antioxidant enzymes activities that scavenge toxic ROS, thereby reducing oxidative damage in plants. SA can also activate protein kinases that phosphorylate and activate transcription factors involved in stress responses. Understanding these mechanisms is essential for developing effective strategies to improve crop resilience in the face of changing environmental conditions. Current information provides valuable insights for farmers and plant researchers, offering new strategies to enhance crop resilience and productivity in the face of environmental challenges. By harnessing the power of SA and its signaling pathways, farmers can develop more effective stress management techniques and optimize crop performance. Plant researchers can also explore innovative approaches to breed or engineer crops with enhanced stress tolerance, thereby contributing to sustainable agriculture and food security.

## Introduction

**Figure 1:**
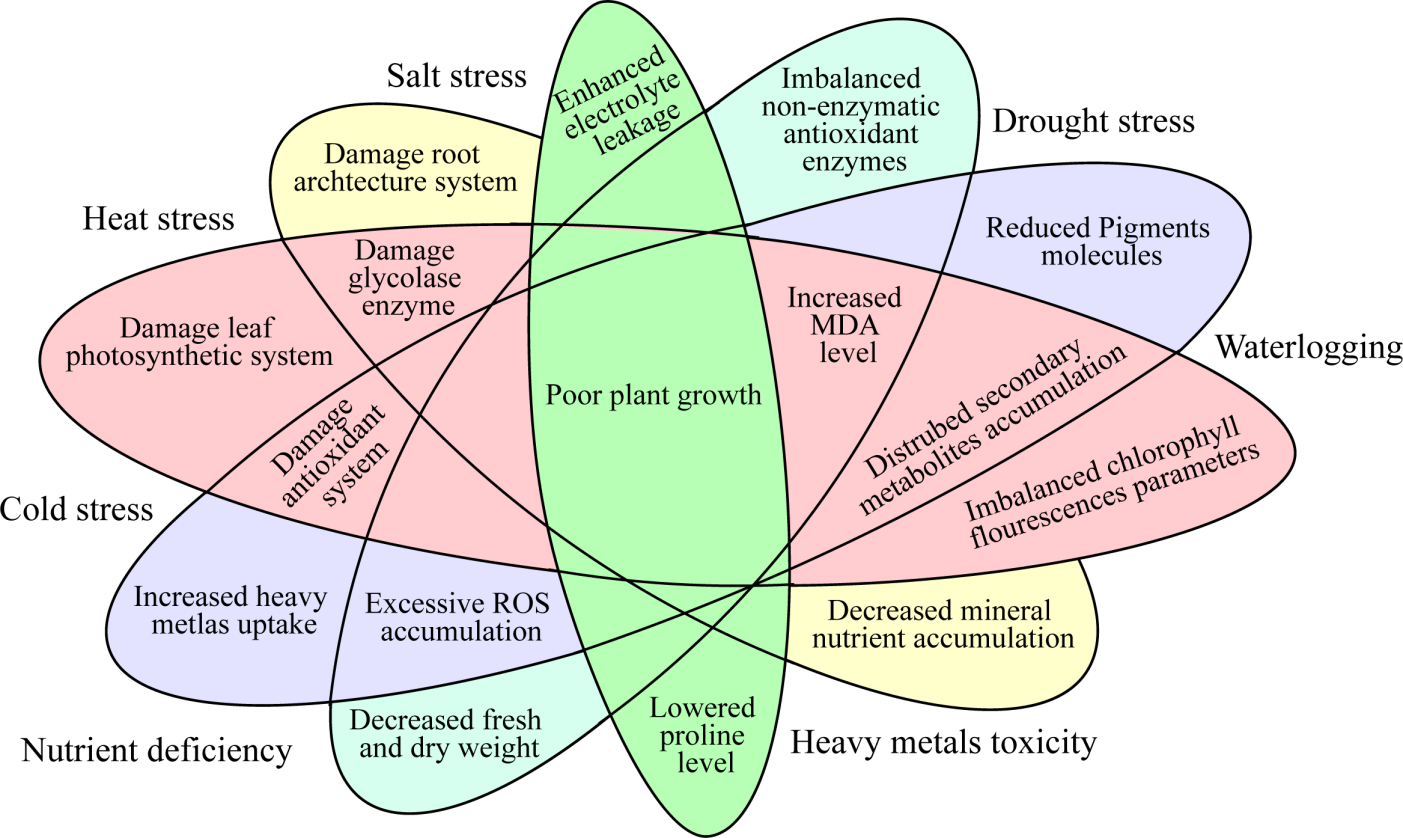
Physiological and molecular responses of the horticultural crops under abiotic stress.

Abiotic stresses are major threats to the production of horticulture crops globally (Kreps et al., 2002). Nevertheless, significant abiotic stresses like heavy metals (HMs), drought, ozone, salinity, ultra-violet radiation, nutrients, temperature fluctuations (low and high), and anthropogenic activities in the developmental period have exacerbated the agriculture system (**Figure 1**) (Khan et al., 2015). From the earliest stage of seed germination through maturity, the imposed abiotic stresses can affect practically all the molecular, physiological, biochemical, and metabolic functions in plants (Jia et al., 2013). This could ultimately result in serious losses in the economic output of horticulture crops (Chaudhry and Sidhu, 2022; Liu et al., 2022). The approach that plants interact with numerous environmental prompts is a furthermost vital question for plant researchers. Drought, heat, and salt stresses are the most prevalent and vital environmental stresses which have adverse effects on plant growth, yield, and quality (Altaf et al., 2022a). Phytohormones are crucial in coordinating several signal transduction pathways under stress environments (Khan et al., 2020). These hormones perform a vigorous function in the plants’ development and play an important part in the plants’ external and internal factors (Ryu and Cho, 2015). Phytohormones are recognized as environmentally acceptable for improving horticulture crops’ tolerance against abiotic stress (Mantri et al., 2012). Plants’ growth is regulated by the phytohormones as they are released in small quantities as a chemical regulator (Altaf et al., 2022b).

SA is the hormone that has been shown to activate several stress-reactive genes. Every aspect of the plant’s life is controlled by the biological substances known as plant hormones containing seed germination and distribution as well as the development of fruit (Klessig and Malamy, 1994). Under abiotic stress tolerance and regulation of the hormones involves in the cascade of the composite signaling from the stimulus sensitivity to the expression of the gene (Souza et al., 2017). According to previous research, the ROS-dependent signaling system and hormone responses interact to allow plants to acclimatize to biotic and abiotic challenges by improving performance (Tognetti et al., 2012).

**Figure 2:**
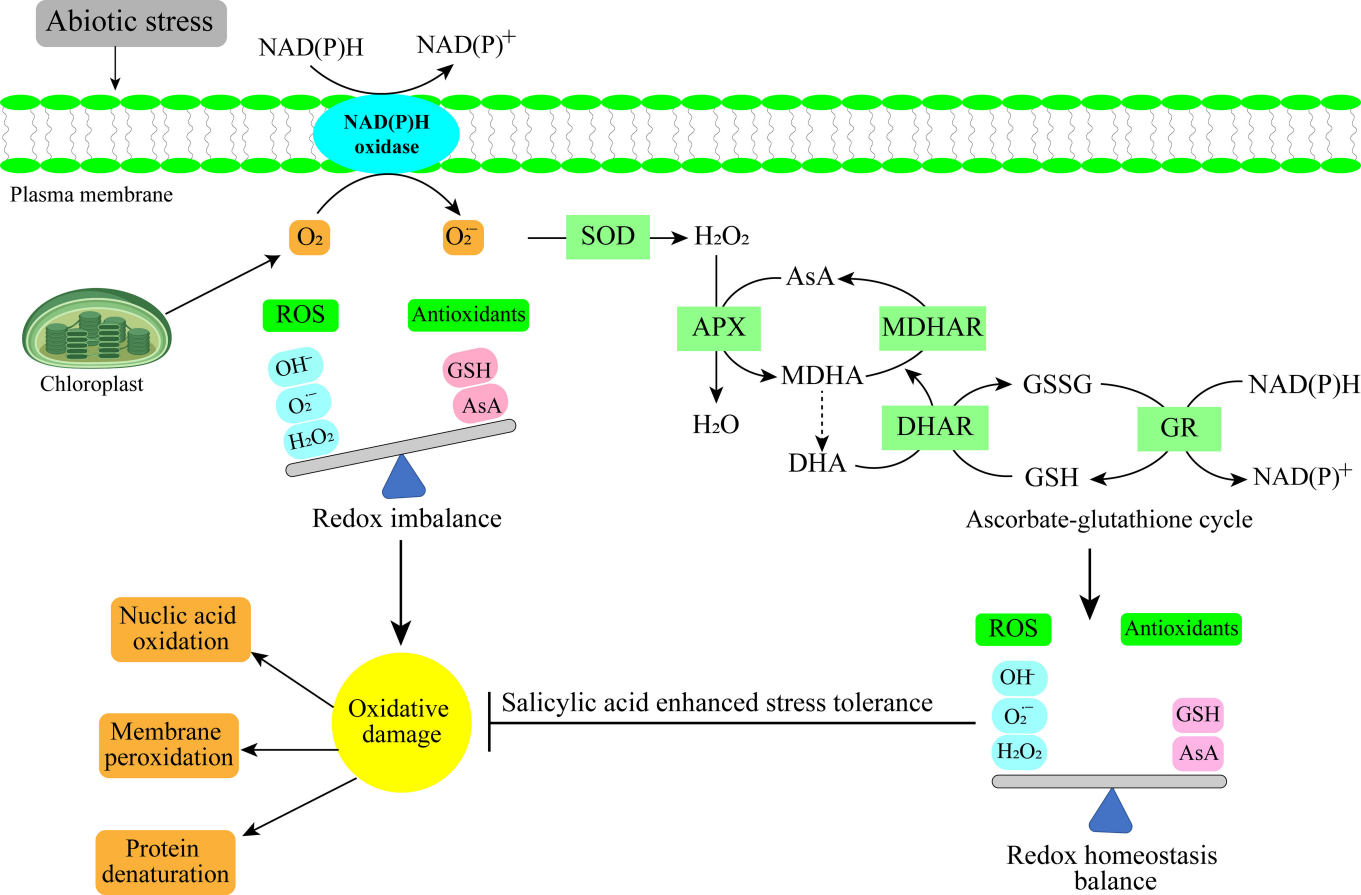
Antioxidant mechanism under abiotic stress in horticultural crops.

It is challenging to exaggerate the importance of SA spray in preventing abiotic stresses on horticultural crops. SA is essential for increasing crop resistance to numerous abiotic stresses. SA causes a series of physiological processes i.e. better water retention, higher antioxidant activity, and reinforced cell walls. These reactions aid the plants in preventing damage brought on by stress, preserving metabolic balance, and boosting the growth (**Figure 2**) (Xu et al., 2015a). SA has a crucial role in protecting horticulture crops, increasing productivity, and maintaining food security in the face of unfavorable environmental conditions by lessening the harmful effects of abiotic stress. The current review aimed to provide a comprehensive understanding of the mechanisms by which SA confers abiotic stress tolerance in horticultural crops and its potential as a tool for improving crop productivity and resilience in the face of changing environmental conditions.

## Overview of SA and its potential as a tool for improving plant growth and development

SA sometimes referred to technically as 2-hydroxybenzoic acid, is an aromatic phenolic molecule produced by plants that contains a hydroxyl group or its functional derivative. Phenolic compounds, particularly systemic acquired resistance (SAR), have a role in trendy activating plant defenses (Vlot et al., 2009). SA improves flowering and fruit set even under normal and stress conditions (Pacheco et al., 2013), promoting root growth, stomatal closure, and reducing transpiration (Gorni and Pacheco, 2016), reversing ABA effects (Davies, 2004), regulating gravitropism, and inhibiting fruit ripening (Srivastava and Dwivedi, 2000). Additionally, it has also been observed that plants of pepper and tomato treated with 05 - 10 µM of SA had increased weight of the dry plant shoots along with the area of the leaf (Sardar et al., 2021).

Nevertheless, on plants growth and gas exchange parameters, the SA stimulatory effects depend on the application technique, exposure time, and ontogenetic plants stages and effective concentrations of the SA vary reliant on species (Horváth et al., 2007; Sardar et al., 2021). SA can indirectly affect flowering because it changes production and plant hormone signaling directions such as auxin, ethylene, and jasmonic acid (Vlot et al., 2009). The exogenous use of SA might cause an uprise in the quantity of endogenous bioactive GA in response, altering the plant’s hormonal condition (Mukherjee and Kumar, 2007). Exogenous application improved the level of phytohormones involved in enhanced plant growth, flowering, fruit set, and ultimately yield (Kim et al., 2009).

SA is the hormone that controls defense of the plants against pathogens and is connected to phenolic chemicals (Houston et al., 2016). To modify the tolerance to different abiotic stimuli, SA is also crucial. SA encourages defensive photosynthetic processes, which leads to regulation in stomatal conductance, carbon dioxide (CO_2_) assimilation, ribulose-1,5-bisphosphate carboxylase/oxygenase activity, and chlorophyll and carotenoid content (Feng et al., 2020). By lowering cellular lipid peroxidation and hydrogen peroxide (H_2_O_2_) buildup, SA application to plants with insufficient water lower cell membrane damage in leaves. Additionally, SA-treated plants have less H_2_O_2_ buildup, which boosts the activity of antioxidant enzymes like catalase (CAT), superoxide dismutase (SOD), and guaiacol peroxidase (GPOD) (Ahmad et al., 2023).

**Figure 3:**
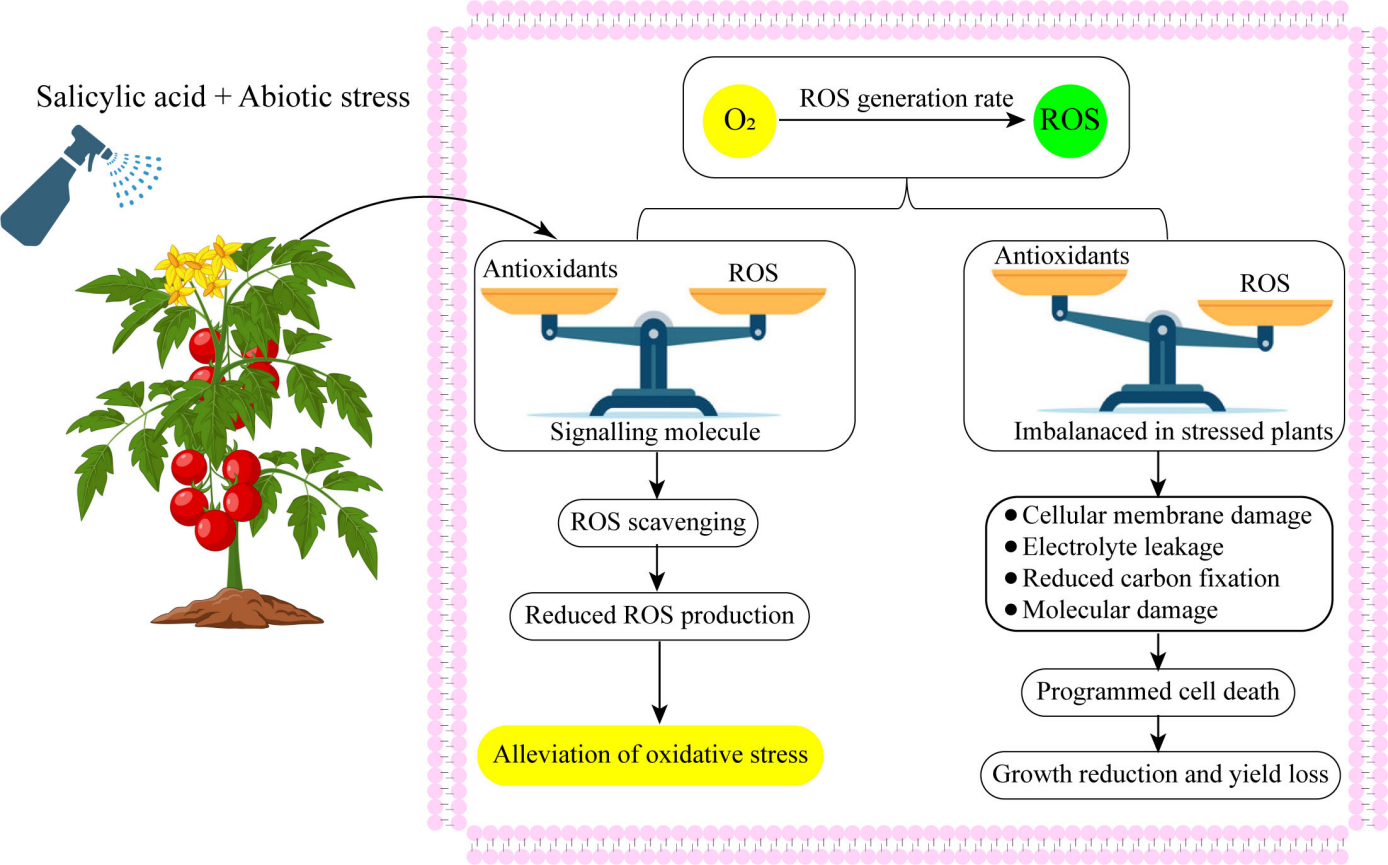
Exogenous salicylic acid application up-regulated antioxidant defense system and balanced redox homeostasis.

SA performs an important part in the plants’ developmental processes (**Table 1**). Research has shown its functions in stomatal conductance, seeds development, glycolysis, the flowering of the plants, production of the fruit, ion transpiration and accumulation (Harper and Balke, 1981; Sardar et al., 2022), and the rate of photosynthesis (Khan, et al., 2003). SA can regulate the antioxidant defenses and reduce oxidative damage in plants (**Figure 3**) (Banu et al., 2009). SA controlled the plant nitrogen, glycine betaine (GB) formation, photosynthesis, proline metabolism, and plant water interactions under abiotic stress (Nazar et al., 2011; Miura and Tada, 2014). Additionally, abiotic stressors have been shown to induce defense-related genes and stress tolerance (Rivas-San and Plasencia, 2011; Kumar, 2014).

**Table 1:** Role of salicylic acid in abiotic stresses in horticultural crops focusing on sustainable productivity.

Stress Name	Plant Name	References
Heavy Metals Stress	Malting Barley (*Hordeum uhulgare* L.)	Song et al. (2014)
Cold Stress	Bean (*Phaseolus vulgaris*)	Orabi et al. (2015)
Drought Stress	*Portulaca oleracea*	Saheri et al. (2020)
Ozone Stress	Strawberry (*Fragaria × ananassa*)	Drogoudi and Ashmore (2000)
UV-B radiation	Pepper (*Capsicum annuum* L.)	Mahdavian et at. (2008)
Cold Stress	Cucumber (*Cucumus sativus* L.)	Hawrylak-Nowak et al. (2010)
Cold stress	Tomato (*Solanum lycopersicum*)	Orabi et al. (2015)
Salinity stress	Guava (*Psidium guajava* L.)	Sharma et al. (2020)
Cold stress	Pepper (*Capsicum annuum* L.)	Korkmaz et al. (2010)
Salinity Stress	Mango (*Mangifera indica*)	Elsheery et al. (2020)
Drought stress	Tomato (*Solanum lycopersicum*)	Hayat et al. (2008)
Drought Stress	Bean (*Vicia faba* L.)	Abdelaal, (2015)
UV-B radiation	Mango (*Mangifera indica*)	Mavrič Čermelj et al. (2021)
Heat stress	Peas (*Pisum sativum*)	Pan et al. (2006)

### SA and drought stress

The most important abiotic stressor limiting crop output is thought to be drought (**Table 2**). Only 10 - 30% of the available irrigation water is used each year (Szilagyi, 2003). The rise of the drought was further aided by the amalgamation of high temperatures and low precipitation (Yu et al., 2013). Global food security is facing a challenge from climate change which pollutes water resources and declining crop production (Hedhly et al., 2009; Gomes et al., 2019). Water shortages would impact agricultural land by up to 50% by 2050 (Hasanuzzaman et al., 2019). Moreover, lack of water is the main environmental barrier to the production of horticulture crops (Ullah et al., 2018; Sirisuntornlak et al., 2019). In arid and semiarid environments, the two most significant environmental stresses drought and salinity occur simultaneously (Slama et al., 2008). Extreme drought and salt stress levels affect a large portion of the earth and harm crop plant output (FAO, 2017). Drought negatively affects plant development growth, and other metabolic functions, and is a limiting factor in the effective production of crop plants worldwide (Osuagwu et al., 2010). The weight of the dry roots can be increased due to the drought stress and also had an impact on nutrient uptake, resulting in a decline in the potassium content of the plant’s roots and shoots.

**Table 2:** Function of salicylic acid application in horticultural plants.

Functions	References
Improved CO_2_ absorption	Saheri et al. (2020)
Increased the plant’s PAL (phenylalanine lyase)	Chavoushi et al. (2020)
Mitigates water deficits	Chakma et al. (2021)
Combat drought problem	Ashraf et al. (2013)
Opening of the stomata	Rai, 2002
Resistance to cold stress	Sayyari et al. (2013)
Osmolytes Contents and Anti oxidative defense system modification	Kohli et al. (2018)
Lipid peroxidation and ROS level decline	Shi and Zhu (2008)
Transpiration and the rate of photosynthesis increases	Khan, et al., (2003)
Controls the plant nitrogen	Nazar et al. (2011)
metabolic and defense pathways	Bücker-Neto et al. (2017)
Antioxidant enzyme activity increase	Kazemi et al. (2010)

Phytohormone supplementation is regarded as an efficient adaptation technique (El-Mageed et al., 2017). SA affects the absorption of ions, the development of the plant, and the transport of substances. It is the crucial plant signaling molecule regarding the plants’ biotic and abiotic stress tolerance (Kazemi, 2014; Gorni et al., 2017). SA treatment had a progressive effect on *Portulaca oleracea* growth and biomass accumulation during water deficit by improving photosynthetic pigments and CO_2_ absorption (Saheri et al., 2020). According to Ahmad et al. (2023), the application of SA on *Carthamus tinctorius* L. during a water shortage increased the plants’ phenylalanine lyase (PAL) enzyme activity and anthocyanin contents but had no impact on the dry matter (Ahmad et al., 2023). Recent findings indicate that foliar SA spray on tomato mitigates water deficits (Chakma et al., 2021). The necessity of verifying SA dosages in scenarios that farmers can use is raised by the fact that grape tomatoes are not the most widely farmed tomato in the world and that the investigational circumstances of this research cannot be reconstructed in the field.

Previous research suggested using SA, which is intricate in the stress signaling compared to abiotic and biotic stresses to combat drought problems (Rabnawaz et al., 2020). SA applied topically to plants stated to cut their susceptibility to stress such as drought and the maximum temperature (Wang and Li, 2006). Opening of the stomata, ion transport regulation, and cleaning of the HMs adverse effects are all functions of SA (Rai, 2002).

### SA and cold stress

Environmental stresses including heat, salinity, cold, and drought harm plant growth and production and cause several physiological, molecular, morphological, and biochemical changes in plants that ultimately cause the plants to become unsustainable (Ahmad and Prasad, 2012). Cold stress inhibits the growth of a plant its development and plant metabolism (Lichtenthaler, 1998). The sensitivity and intolerance of many crops to low temperatures (cold stress) have an impact on crop yield. A sudden temperature change harms the plants (Schwartz et al., 2006). According to Ruelland and Zachowski, (2010), plants typically succumb to chilling and freezing stress as a result of membrane damage, change in enzyme activity, necrosis, changes in cytoplasm viscosity, and chlorosis.

Low-temperature stress is most dangerous for vegetable crops regarding their productivity and quality (Yadav, 2010). The low temperature of about 0 - 15 °C is vulnerable for summer vegetables, for the whole plant life cycle including germination of seeds, vegetative growth, and reproduction. Cold stress plants reproductive stages, cause them to blossom later and produce sterile pollen, which has a significant negative impact on agricultural productivity (Suzuki et al., 2008; Yadav, 2010). Among abiotic stresses, the low temperature has a significant impact on the existence and geographic spread of the plants, as well as on the agricultural production of plants in mountainous terrain. Disruptions to plant growth and productivity lead to significant crop failure (Hatfield and Prueger, 2015). Low-temperature initial effect on the plant is the membrane fluidity decrease may be the harm perception of the potential site (Orvar et al., 2000). ROS damaged the membrane due to freezing temperature with cell wall and membrane adhesions and causes the rupture of the cell (Olien and Smith, 1997).

SA application enhanced the number of fruits in cucumber and pepper (Elvwan and Hamahyomy, 2009; Hayat et al., 2010). SA applied on the cucumber leaves enhanced their resistance to cold (Sayyari et al., 2013). Lower acetyl SA and SA concentrations (0.1 mM and 0.5 mM) have been revealed to be highly effective against low-temperature stress in bean and tomato (Orabi et al., 2015).

### SA and heat stress

When plants are subjected to abiotic stress, they cannot relocate to more hospitable conditions, which has a substantial adverse effect on the growth of the plant, development, and yield (Lippmann et al., 2019). High temperatures (HT) are a significant stress, and in recent decades, global warming has increased the rise in air temperatures (Lippmann et al., 2019). The processes by which plants adapt to extreme temperatures are therefore quite interesting. One of the most harmful pressures among the continually shifting environmental factors is the steadily rising ambient temperature. By 2100, temperatures are expected to be 1.8 – 4.0°C greater than they are now due to the global air temperature increase of 0.2°C every decade (IPCC, 2007). Plants that are subjected to heat stress (HS) experience significant, and occasionally fatal, negative impacts. Plants have developed sophisticated HS response mechanisms to deal with such circumstances. HS influences many essential physiological functions of plants, including photosynthesis, water metabolism, and respiration. Characterizing HS reactive genes, non-coding RNAs, DNA methylation, and histone amendments have made significant strides (Ruelland and Zachowski, 2010).

For plant scientists, developing new crop cultivars resistant to HT is a big task (Zhang et al., 2006; Moreno and Orellana, 2011). HT affects plants in numerous means, depending on their intensity, duration, the kinds of plants present, and other environmental conditions (Rodríguez et al., 2005; Wahid et al., 2007). However, the features that confer resistance to HT have yet to be identified and confirmed. Researchers studying plants under HT stress are attempting to understand the plant retorts that result in heat tolerance as well as how plants might be controlled in HT situations. Omics techniques and the development of transgenic plants by modifying target genes are two recently highly explored molecular approaches (Schoffl et al., 1999; Kosová et al., 2011; Duque et al., 2013). Investigating these underlying molecular mechanisms may help to develop stress-resistant crop variants and enable the HT cultivation of vital crop plants.

SA has a role in altering resistance in plants against biotic stress and abiotic stress due to the part that plays in immune system activation (Kawano and Bouteau, 2013). Researchers are very interested in the use of SA to enhance plant thermo tolerance as well as the physiological and biochemical mechanisms involved. SA foliar spraying promoted heat tolerance in essential horticultural plants like cucumbers and grapes (Wang et al., 2010; Yang et al., 2022). By controlling enzymatic activity, SA application boosts the rate of photosynthesis and preserves cell membrane stability (Waqas et al., 2021). The formation of cardenolides and antioxidants is also stimulated by SA treatments, which may be crucial in boosting resistance to HT stress (Zhang et al., 2020).

### SA and salinity stress

Salinity had numerous detrimental effects on the germination of seeds, plant development, and crop yield (Munns and Tester, 2008). Various irrigated regions get salinized, primarily as a result of the usage of saline water. Approximately, 45 million hectares of land are damaged due to salinity (Munns and Tester, 2008). Plants are impacted by high salinity in a variety of aspects, including oxidative stress, water stress, nutritional issues, metabolic process changes, ion toxicity, decreased expansion and cell division, membrane disruption, and genotoxicity (Hasegawa et al., 2000; Munns, 2002; Zhu, 2007). These effects collectively by inhibiting the survival of the plant, its growth, and its development (Sardar et al., 2023).

A major factor restricting agricultural production is salinity (Slama et al., 2008). It will be difficult to feed the 9.3 billion people on the planet in 2050. According to estimates, agricultural production must increase by more than 60% from 2005 to 2007 levels by 2050. (Fita et al., 2015). However, stress factors like salinity are a constant danger to effective agricultural productivity. Climate change, groundwater penetration, seawater backflow, and human activities like fertilizer application and irrigation have all contributed to an increase in soil salinity in recent years (Arani et al., 2023). The salinity of the soil reduces plant product quality, productivity, and yield (Yang and Guo, 2018a). Salinity stress affects the land by about 950 million hectares, although the percentage of soil salinity is rising in the future (Yang and Guo, 2018b).

When salt stress initially appears, excessive salt and chloride ions restrict root water because it decreases the water potential surrounding the roots of the plants (Negrão et al., 2016). Second, too much salt and chloride in plants cause toxicity of the ion. It not only interferes with the homeostasis of ions like Na^+^ and K^+^ but also averts the efficient uptake of nutrients like Ca^2+^, leaving plants deficient in vital nutrients (Zhu, 2002; Wu, 2018). Plants experience oxidative stress due to the overabundance of ROS brought on by osmotic and ionic stressors (Zhu, 2016). In chloroplasts and mitochondria, excessive H_2_O_2_ accumulation impairs respiration and photosynthesis under salt stress (Balal et al., 2011).

To address the salt issue, a variety of phenolic compounds and plant growth regulators (PGRs) are used to decrease their effects. SA is an endogenous signal molecule that functions in plants’ ability to withstand environmental challenges. Plants must be able to respond to abiotic and biotic stresses (Moustafa-Farag et al., 2020). SA also promotes several physiological processes important for plant growth (Basit et al., 2018; Guo et al., 2022). Antioxidant SA is regarded as having hormone-like properties. Abiotic stressors can raise the SA concentration in the plant, strengthening its ability to withstand these challenging circumstances (Yuan and Lin, 2008). SA is essential for stomatal conductance, photosynthesis, and transpiration. Additionally, it decreases Na^+^ and Cl^-^ buildup in plant tissues, enhancing antioxidant defense (Sardar et al., 2022). SA can thereby increase salt tolerance, reducing the adverse effects that occur on plants (Manaa et al., 2014; Semida et al., 2014). The upregulation of anti-stress mechanisms and the restoration of the development process are two examples of SA’s beneficial effects (Mahmood, 2006).

### SA and heavy metals

HMs have surpassed pesticides and eminent pollutants like sulfur dioxide (SO_2_) and CO_2_ (Chen et al., 2007). It is expected that HMs could overtake solid waste and nuclear waste as the most harmful contaminant (Lajayer et al., 2017). Eating is the primary way that 70% of all HMs and their derivatives enter human bodies (Jaishankar et al., 2014). HMs are emitted into the air, land, and water from numerous sources and as a result of human activity (Tchounwou et al., 2012). HMs toxicity has grown to be a significant issue in many terrestrial ecosystems all over the globe. Recently, excessive industrialization damages soil quality and agricultural yield by collecting HMs (Shahid et al., 2015).

Changes in the soil pH which damage the texture of the soil, the presence of various elements, and the HMs accumulation, may delay a variety of molecular as well as plant physiological processes, which may either directly or indirectly limit plant development (Panuccio et al., 2009). Several developmental and biological processes need HMs such as Cu, Mo, Ni, Mn, Co, and Zn at very low amounts (Salla et al., 2011; Shahid et al., 2015). However, when their concentration exceeds suboptimal levels, these metals along with four other extremely poisonous heavy metals, Be (beryllium), As (arsenic), Al (aluminum), Cd (cadmium), Pb (lead), and Hg (mercury) can significantly decrease the crop yield (Pierart et al., 2015). These harmful substances result in aberrant plant morphology and metabolic issues that lower plant production (Amari et al., 2017). ROS such as OH^-^, O_2_^-^, and H_2_O_2_ are produced as a result of these irregularities, which disturb the redox balance of the cells (Ibrahim et al., 2015; Shahid et al., 2015). This redox state imbalance is known to have a significant role in the HMs toxicity in plants. Earlier studies showed that HMs accumulation in horticulture products harms human health (Uzu et al., 2011; Shahid et al., 2015).

Metal absorption and sequestration capabilities vary across plant types and plant regions. There are considerable changes in the absorption and transport of metals across species of plants and numerous varieties of the same species plant species (Shahid et al., 2017). The majority of cereal crop plants’ roots are located 25 cm below the surface of the soil, where the plant absorbs HMs. Metals like Cd and Pb are primarily collected in roots, which are the major target for metal accumulation (Kumar et al., 2012). The cellular transport system is one method that plants have progressed to lessen the toxicity of the metal ion.

Phytohormones are small signaling molecules that have a significant impact on virtually every aspect of plant development and growth. Distinct hormones can have quite different mechanisms of action for various functions. So, although several hormones may be involved in the regulation of a single function, this is shown that a single hormone may govern the range of developmental and cellular processes (Gray, 2004). Phytohormones such as IAA (indole-3-acetic acid), also known as auxin, abscisic acid (ABA), cytokinin (CK), gibberellin (GA), ethylene (ET), jasmonic acid (JA), brassinosteroids (BRs), cytokinin (CK), salicylic acid (SA) and the most recent discovered strigolactones (SLs) are essential for plant growth and development, signaling, and crosstalk. Phytohormones are much important for plants to protect them from biotic stress as well as abiotic stress (Nishiyama et al., 2011; Colebrook et al., 2014; Xu et al., 2016; Bücker-Neto et al., 2017). In agronomical crop management strategies, phytohormones have been used as regulators of HM absorption to reduce metal toxicity (Piotrowska-Niczyporuk et al., 2012). Exogenous phytohormone application demonstrated to be safe to employ and to produce favorable outcomes for plants exposed to HM toxicity (Zhu et al., 2012; Zhu et al., 2016; Masood et al., 2016). Phytohormones like IAAs, BRs, SAs, CKs, GAs, and much more play important signaling roles, metabolic and defense pathways, but the way they alleviate HM stress is at this time a subject of great attention worldwide (Bücker-Neto et al., 2017). Phytohormone priming is carried out to promote imminent research for crop stress treatment.

Under HM stress, naturally accruing SA phenolic molecule is associated with the defense response in plants (**Table 3**). SA contributes to coordinating plant development, ripening, and abiotic stress responses (Rivas-San and Plasencia, 2011). Both the ABA and SA are engaged in managing the HMs response (Miura and Tada, 2014). The presence of SA can also control a plant’s tolerance to salt, heat, and cell death under heavy metals toxicity (Naz et al., 2022). SA shown to activate the antioxidant defense system and regulate Cd detoxification pathways, hence reducing Cd toxicity. Cd exposure increases barley SA content (Hordeum vulgare) roots. SA and other phytohormones, such as JA and ET, commonly interact with one another (Jia et al., 2013). Plant stress response as a part of hormone production, transport, and storage result in a cascade of signaling pathways (Matilla-Vazquez and Matilla, 2014). ET biosynthesis enzyme activity increases in the presence of HM, and MAPKs phosphorylate ACS2 and ACS6 (Skottke et al., 2011; Bücker-Neto et al., 2017; Ahmad and Anjum, 2023).

**Table 3:** SA enhanced HMs stress in different Horticultural plants.

Plant Name	Heavy Metal	Plants Adaptive Reaction	Reference
Cucumber	Mn	Lipid peroxidation and ROS level decline. Antioxidants (Glutathione and Ascorbate) increase	Shi and Zhu (2008)
Peanut	Cd	Metal Toxicity alleviation. Photosynthesis, Mineral nutrients, Growth, and Chlorophyll enhancement	Xu et al (2015a; 2015b)
Onion	Pb	Minimize the DNA damage, Increased the CAT (Catalases), APX (Ascorbate), SOD (Guaiacol Peroxidases), and GPX (Superoxide Dismutases)	Kaur et al. (2021)
Spinach	B	Decreased the Lipid peroxidation, H_2_O_2_ activity, and MDA contents, While increasing the Chlorophyll contents, CAT, and SOD activity.	Eraslan et al. (2008)
Tomato	Cd	Enhanced the plant leaves, stem and root biomass, and Photosynthetic rate	Wei et al. (2018)
Cucumber	Mn	Reduced the Mn transport from roots to the plant above parts, Lipid peroxidation, ROS contents, APX, and CAT activities. Increased the Ascorbate, SOD, GR (Glutathione reductase), POD, and DHAR (Dehydroascorbate reductase).	Shi and Zhu (2008)
Pea	B	Enhanced ROS production and degradation, Plant fresh and dry biomass, Carotenoids, and Chlorophyll contents. Reduction of the H_2_O_2_ and MDA contents.	Oliveira et al. (2020)
Potato	Cd	Increased the antioxidant defense system, RWC (relative water contents), and chlorophyll and proline. While decreasing the O_2_^-^ (Superoxide Anion redicle), MDA (Malondialdehyde), and H_2_O_2_ (Hydrogen Peroxide).	Li et al. (2019)
Lemon balm	Ni	Improved the plant fresh and dry shoot and root biomass, Carotenoid contents, and decline in the electrolyte leakage.	Soltani Maivan et al. (2017)
Safflower	Zn	Reduced oxidative stress activity. Increased in the ASC (Ascorbate) level, PC (Phytochelatin), GSH (Glutathione)	Eisalou et al. (2021)
Cucumber	Cu	Minimized the GSH, Proline, and MDA contents. Increased plant growth.	Çanakci-Gülengül et al. (2017)
Lettuce	Cd	Enhancement of proline contents, Peroxidase enzyme activity, and ascorbic acid,	Talabany and Albarzinji (2023)
Pepper	As	Increased glutamine synthetase, Nitrogen metabolism, Nitrate reductases, and Glutamate synthase activity.	Kaya et al. (2022)
Periwinkle	Ni	Improvement of Alkaloid contents, Vincristine, and anticancer alkaloids vincristine	Idrees et al. (2013)

### SA and ozone stress

Tropospheric ozone (O_3_) is a naturally occurring element of the atmosphere; however, since industrialization, anthropogenic emissions of NO (nitrogen oxides), NMVOCs (Non-methane volatile organic compounds), CO (Carbon monoxide), and CH_4_ have caused the O_3_ to be produced as a result of photochemical reactions. O_3_ levels have increased as a result, rising from pre-industrial levels of less than 20 ppb to contemporary levels, which have boosted the average of Northern Mid-Latitude O_3_ concentrations (30-50 ppb) (Cooper et al., 2014). Since the 1950s, mean O_3_ levels have been increasing, with the Northern Hemisphere (NH) seeing an increase of 1 to 5 ppb/decade and the Southern Hemisphere (SH) seeing an increase of 2 ppb/decade (Cooper et al., 2014). Over the past 50 years, evidence of O_3_ harm and damage to vegetation as a result of O_3_ phytotoxicity has increased significantly along with these concentrations (Grulke and Heath, 2020). O_3_ is a secondary pollutant, so its concentrations vary a lot in space and time. This is because of things like the season, interactions between land and coast, monsoon systems, and the exchange of air between the stratosphere and troposphere. Accordingly, in semi-rural and rural areas O_3_ concentrations are typically maximum that are downwind of the industrial and the urbanization sources of precursor emissions, i.e., in areas vital to agriculture, forestry, and grasslands and the ecosystem services they provide (Mills et al., 2013). When O_3_ degradation (via chemical transformation and deposition) is slower than O_3_ creation, background O_3_ concentrations will be increased. O_3_ and its precursors are also carried around the planet in air masses. The duration, frequency, and magnitude of O_3_ levels are all crucial factors in determining how plants will react, and these global processes have an impact on local to regional O_3_ concentrations.

Limited research has been done on the ozone effects on plant reproductive characteristics. Leisner and Ainsworth (2012) examined 128 peer-reviewed studies to assess the detrimental effects of present ozone levels on the reproductive development and production of several crops. However, current levels of ambient ozone considerably reduced the number of seeds (16%), fruits (9%), and fruit weight (22%). The development and makeup of fruits vary with the growing environment and may be impacted by other environmental factors. Ozone can affect reproductive development at many distinct stages (Black et al., 2000). It was demonstrated that strawberry fruiting plants bare to ozone (184 g/m^3^ O_3_ for 69 days) experienced an initial acceleration of inflorescence formation, monitored by a decrease in inflorescence production rate and fruit setting rate (Drogoudi and Ashmore, 2000). Moreover, ozone treatment (156 g m^3^ ozone for 2 months of exposure) reduced ascorbic acid content, increased peroxidation of lipids, and decreased strawberry fruit taste because it altered the fruit’s carbohydrate pool (Keutgen and Pawelzik, 2008; Yi et al., 2021). This research showed that ozone modifies the development of reproductive organs to influence fruit yield and quality. Consumers’ demands for higher-quality fruit have grown.

In response to relating the ozone effects to concentrations averaged at one-hour intervals, which might serve as a substitute for dosage, it is crucial to summarize concentrations in a physiologically significant way (Lefohn, 1992). The exposure index should, in theory, be based on the idea of effective dose (Runeckles, 1974), that is, it should capture the exposure features that are most closely related to the quantity of ozone that is absorbed by the plants. In this case, the absorbed dosage would be the integral of the rate of absorption (flux) over time, which could be determined for ozone by dividing the concentration at the leaf surface by the leaf conductance (Fowler and Cape, 1982).

The conduction of the atmosphere could be incorporated into this conception (Grunhage and Jager, 1994), when there is adequate air mixing (high air conductivity), ozone concentration, and conductance of the leaf control the diurnal pattern of ozone flux. In open-top contact chambers, this is the case. In crops, the use of radiation as a substitute for leaf conductance has been proposed due to the lack of data on leaf conductance (Rawlings et al., 1988). It is necessary to characterize exposure by using ozone concentrations during the daylight hours measured (for example, >50 W/m^2^ global radiation). However, no such distinction should be established for species that exhibit significant leaf conductivity at night. Although it is also known that other parameters, such as air humidity, soil water availability, and temperature affect leaf conductance, these factors have not yet been employed in long-term research to quantify ozone uptake or dose.

### SA and UV-B radiation

Sunlight, the supreme energy source on Earth, is required for all vital biological activities. On the earth’s surface, UV light is one of the principal solar spectrum components (Dotto et al., 2018). The geomagnetic field and stratospheric ozone layer are only two examples of the many natural barriers that exist to lessen the effects of hazardous natural radiations on biotic components (Herndon et al., 2018). Approximately, 90% of (290-320 nm) of UV-B and 100% of (100-290 nm) light cannot reach the Earth’s surface due to the stratospheric ozone layer. Although over the past two decades, the thinning of the stratospheric ozone layer has gained attention due to the massive buildup of environmental pollutants that eventually cause UV-B rays to reach the earth’s surface (Barnes et al., 2019; Neale et al., 2021). As a result, concern over UV-B radiation’s effects on plants has increased due to its increased penetration.

**Figure 4:**
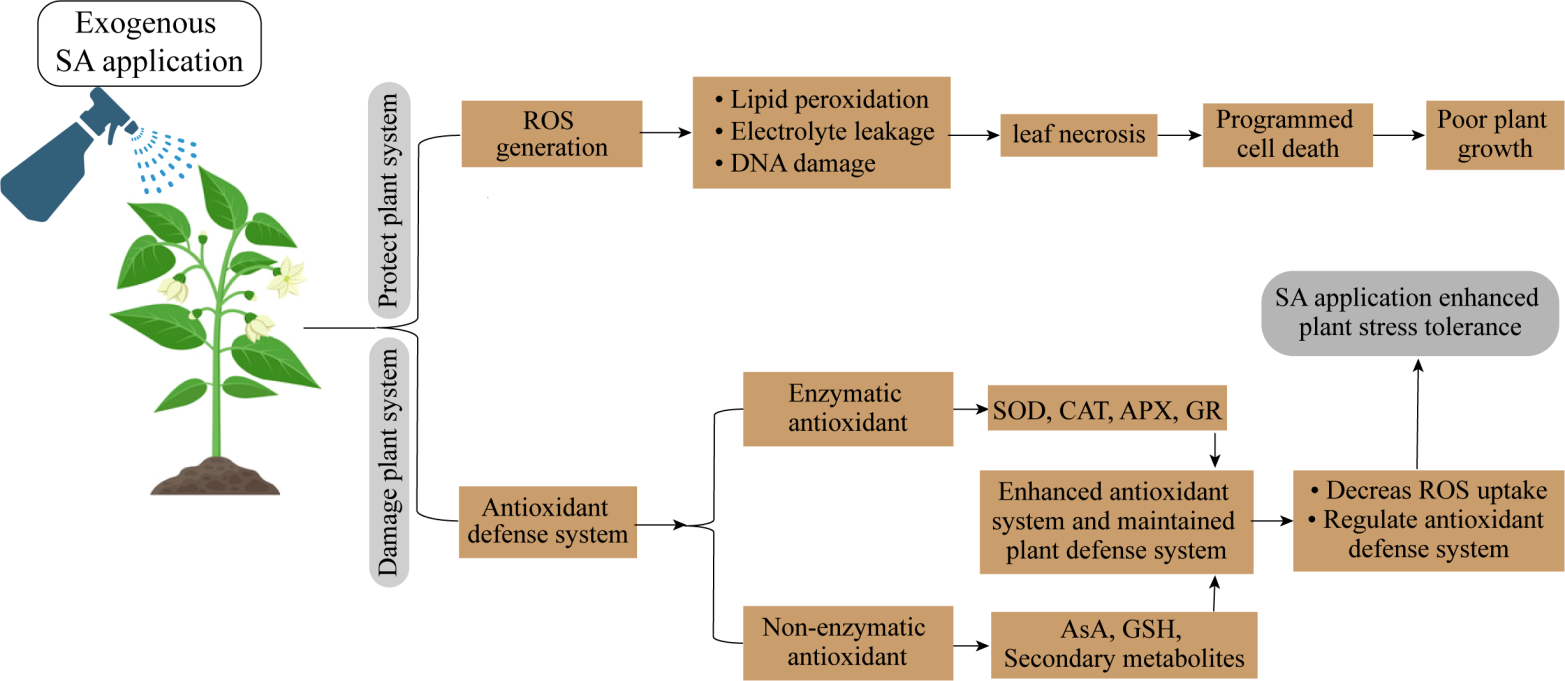
Salicylic acid supplementation enhanced abiotic stress tolerance in horticultural crops.

UV-B-induced morphological alterations in plants may have an impact on how well they compete for light. Deformed morphological characteristics are the result of UV-B radiation’s harmful impacts. UV-B exposure decreased plant leaf area, height, and dry weight while increasing auxiliary branching and leaf curling (Greenberg et al., 1997; Furness et al., 1999). After a few weeks of UV-B exposure, the leaf area, and plant dry weight of rice plants significantly reduced. Weih et al. (1998) found that improved UV-A decreased the amount of leaf area per unit plant biomass (leaf area ratio), but increased the amount of biomass produced per unit leaf area (leaf area productivity) and per unit leaf nitrogen (leaf nitrogen productivity). It was clear that these variables affected the relative growth rate and nitrogen productivity since high UV-B levels decreased the leaf area ratio, leaf area productivity, and leaf nitrogen productivity (Manzoor et al., 2023). The principal causes of UV-B-induced the loss of photosynthetic pigments, thylakoid membrane integrity, reduced rubisco enzymatic activity, and the down-regulation of photosynthesis-related gene transcript levels (Jansen et al., 1996; Hollósy, 2002). Adverisites occur from abiotic stress tolerance can be mitigated by SA application (**Figure 4**; **Table 4**).

**Table 4:** Effects of Salicylic acid use in different abiotic stresses in the plants.

Plant name	Stress Type	Antioxidant Enzyme	Non-enzymatic antioxidant	Stress biomarkers	References
Tomato	Salinity	APX decreased and GPX increased	Damage DNA, inactivate enzymes, and cause lipid peroxidation	H_2_O_2_ increased	Monteiro et al. (2011)
Tobacoo	Ozone	antioxidant enzyme decreased; enzyme BAZ-hydroxylase increased	Photosynthesis decreased	Antioxidant enzymes (CAT and SOD) decline	Yalpani et al. (1994)
Bluegrass	UV-B Radiation	Antioxidant enzymes (CAT and SOD) decreased	Inhibition of photosynthesis, premature senescence, and altered biomass partitioning	Anthocyanin and a-tocopherol contents decreased	Ervin et al. (2004)
Pea	Heat stress	MDA contents decreased	Promote plant thermotolerance, PAL, and BA2H.	Induce the synthesis of Hsp (Heat shock protein)	Pan et al. (2006)
Cucumber	Cold Stress	Benzoic acid 2-hydroxylase activity increased	Significantly decreased the shoot biomass	Reduced the H_2_O_2_ accumulation	Dong et al. (2014)
Spinach	Salinity	Reduction of electrolyte linkage	Enhanced root and shoot length and dry weight of the plant	Increased Chlorophyll a and b and total chlorophyll contents	Gilani et al. (2020)
Potato	HMs	Declined the MDA contents	Increased the RWC (relative water contents), Chlorophyll, and Proline	Enhanced the ROS metabolism, SOD, CAT, EC, GR, and APX contents	Li et al. (2019)
Carrot	Salinity	Enhanced the total antioxidant activity	Increased the root’s fresh and dry weight	Regulation of B and Cl ion and Proline	Eraslan et al. (2007)
Cucumber	Heat Stress	Increased the CAT and SOD activity	Maximize the Photosystem II photochemical reactions	Decreased the H_2_O_2_ and Thiobarbituric acid reactive substances.	Shi et al. (2006)
Pepper	Heat Stress	Decreased the ROS and Increased the protective enzymes activity	Maintain root vigor and cell structure integrity. Also, inhibit the water boss	Reduced the oxidative damage	Zhang et al. (2020)
Common Bean	HMs	Increased the Catalase, Peroxidase, Superoxide dismutase and antioxidant enzymes, and proline	Improvements in photosynthetic pigments, Nitrogen contents, and growth traits	Decreased the electrolyte leakage and mineral ions uptake	Khalil et al. (2021)
**Mung Bean**	UV B Radiation	Reduce the Superoxide dismutase, peroxidase, and Catalase	Increased the chlorophyll contents an	Enhanced the Protein contents and carotenoids	Bano and Singh (2017)
**Onion**	Drought stress	Reduce the osmoprotectants	Increased the RWC, Photosynthetic activity, and membrane stability index	Increased Chlorophyll a contents	Semida et al. (2017)
**Garlic**	Salinity	Increased the bulb diameter and nick bulb diameter	Increase the average clove weight, bulb diameter, number of cloves per bulb, and bulb fresh weight	Enhanced the potassium concentration	Shama et al. (2016)

## Conclusion and future horizon

**Figure 5:**
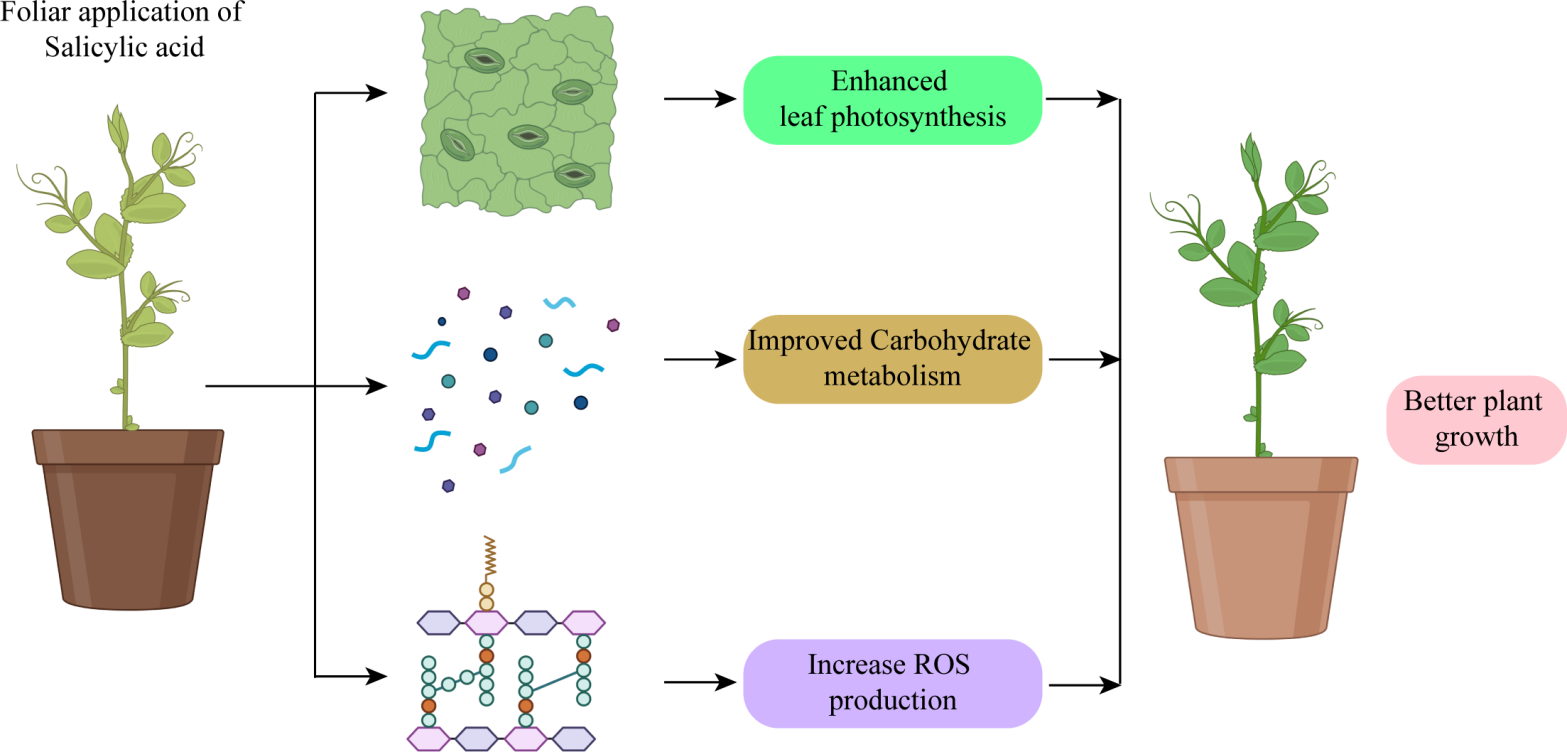
Salicylic acid application protected photosynthetic system and promoted plant growth.

SA works as a signaling molecule and regulates physiological activities in plants such as photosynthesis, metabolic, and growth processes (**Figure 5**). Numerous studies indicate that SA plays a significant part in regulating how plants react to different abiotic stressors. Depending on the plant type, the SA was proven to be efficacious in various foliar sprays/incorporation with growing media treatment forms. SA helps crops withstand the effects of salinity, cold, heat, heavy metals, and drought stressors. Foliar SA treatment proved effective in reducing the negative effects of water deficiency on plants. By boosting the activities of antioxidant enzymes, SA administered to drought-stressed seedlings improved physiological traits and decreased ROS generation, which lessened drought stress and increased plant growth. One method to reduce the low-temperature stress on crops is the application of PGRs and the unitization of genetic tools and plant breeding. PGRs are more important in increasing resistance to cold stress. There has been a lot of research on the use of exogenous SA to reduce heat exhaustion.

Future research should focus on how SA controls cross-talk with PGR operating at high, medium, and short ranges (auxins, gibberellins, cytokinins, ethylene, etc.). It will be possible to identify plant systems devoted to maintaining an optimum balance between growth and defense with further study into the double roles in the development and stress response of salicylic acid. Moreover, this innovative growth regulator has prodigious potential as a running tool for allowing crops to attain greater yields.

## Author contributions

HY and RF: Conceptualization, Literature survey, Writing major original draft, Review structure. LL and WY: Literature survey, Writing- review and editing, Figure designing. QH: Literature survey, Writing- review and editing. CY: Reviewing and editing, References collection. HY: Supervision, WH and WG, JW: Preparing figures and tables: All authors contributed to the article and approved the submitted version.
